# Mechanistic Insights into the Anticancer Potential of *Asparagus racemosus* Willd. Against Triple-Negative Breast Cancer: A Network Pharmacology and Experimental Validation Study

**DOI:** 10.3390/ph18030433

**Published:** 2025-03-19

**Authors:** Arif Jamal Siddiqui, Salem Elkahoui, Ahmed Mohajja Alshammari, Mitesh Patel, Ahmed Eisa Mahmoud Ghoniem, Randa Abdeen Husien Abdalla, Hemlata Dwivedi-Agnihotri, Riadh Badraoui, Mohd Adnan

**Affiliations:** 1 Department of Biology, College of Science, University of Ha’il, Ha’il P.O. Box 2440, Saudi Arabia; s.elkahoui@uoh.edu.sa (S.E.); ah.alshammari@uoh.edu.sa (A.M.A.); a.ghoniem@uoh.edu.sa (A.E.M.G.); badraouir@yahoo.fr (R.B.); drmohdadnan@gmail.com (M.A.); 2 Research and Development Cell (RDC), Parul University, Waghodia, Vadodara 391760, Gujarat, India; patelmeet15@gmail.com; 3 Department of Biotechnology, Parul Institute of Applied Sciences, Parul University, Waghodia, Vadodara 391760, Gujarat, India; 4 Department of Clinical Laboratory Sciences, College of Applied Medical Sciences, University of Ha’il, Ha’il P.O. Box 2440, Saudi Arabia; r.abdalla@uoh.edu.sa; 5 Department of Biophysics, University of Delhi, South Campus, New Delhi 110021, India; hemlata.lifescience@gmail.com

**Keywords:** *Asparagus racemosus*, triple-negative breast cancer, molecular docking, apoptosis, cell cycle arrest, in vitro analysis

## Abstract

**Background/Objectives**: The present study investigated the anticancer potential of *Asparagus racemosus* Willd. against triple-negative breast cancer (TNBC) using a combined in silico and in vitro approach. **Methods**: Network pharmacology identified 115 potential targets shared between *A. racemosus* phytochemicals and TNBC, highlighting key cancer-related pathways. Molecular docking predicted strong binding affinities between specific phytochemicals (beta-sitosterol, quercetin, and others) and crucial TNBC targets, including AKT1 and ERBB2. **Results**: Molecular dynamics simulations validated these interactions, demonstrating stable complex formation. In vitro, *A. racemosus* crude extracts exhibited potent anticancer activity against MDA-MB-231 TNBC cells, showing a dose-dependent reduction in viability (IC_50_ = 90.44 μg/mL), induction of G1 phase cell cycle arrest, and significant early apoptosis. **Conclusions**: These integrated findings provide compelling evidence for the anticancer potential of *A. racemosus* against TNBC, suggesting its promise for further development as a therapeutic strategy.

## 1. Introduction

Triple-negative breast cancer (TNBC) poses a considerable therapeutic challenge in oncology due to the lack of estrogen, progesterone, and HER2 receptors, which restricts treatment options and often results in unfavorable outcomes [1]. Therefore, the development of innovative and effective treatments for this challenging cancer remains a critical priority. Natural products, especially those sourced from medicinal plants with a history of therapeutic use, represent a promising area of investigation in this pursuit. Among these, *Asparagus racemosus*, commonly known as Shatavari, is notable for its wide range of pharmacological properties, encompassing antioxidant, anti-inflammatory, and anticancer effects [2,3].

*Asparagus racemosus* is a perennial climbing plant belonging to the Asparagaceae family, extensively acknowledged in traditional Ayurvedic medicine for its diverse therapeutic properties. This plant is recognized for its adaptogenic, galactagogue, and phytoestrogenic effects, making it particularly valuable in reproductive and women’s health contexts. Notably, the roots contain important phytoconstituents, such as steroidal saponins, particularly shatavarin I-IV, which are touted for their immunomodulatory effects and ability to enhance lactation [3,4,5]. Beyond reproductive health, *A. racemosus* has exhibited efficacy in various physiological and therapeutic applications, including antioxidative, anti-inflammatory, anti-anemic, and cardioprotective properties [6,7,8].

The medicinal potential of *A. racemosus* can be attributed to its rich composition of bioactive compounds. Research highlights its capacity to modulate lipid profiles, where its extraction leads to decreased LDL and VLDL cholesterol levels, therefore supporting cardiovascular health [7]. In experiments, the plant has effectively reduced oxidative stress markers, demonstrating significant antioxidant activity, especially in its alcohol extracts, which is posited to be due to its high concentrations of flavonoids and polyphenols [8,9]. Furthermore, *A. racemosus* has been studied for its benefits in managing conditions such as anemia and thrombocytopenia, owing to its array of phytoconstituents, including alkaloids and saponins, which promote the formation of red blood cells [6,10].

The versatility of *A. racemosus* extends to its application in modern medical research, where it has shown potential as a therapeutic agent in combating various conditions, from gastroprotective to neuroprotective activities. Its traditional uses for treating ailments, such as diabetes, inflammatory diseases, and even certain cancers, are corroborated by contemporary studies documenting its multifaceted pharmacological potential [2,11]. This plant contains a diverse array of bioactive compounds, including steroidal saponins (shatavarins I–IV), flavonoids, alkaloids, polyphenols, and essential oils, which are believed to contribute to its therapeutic effects [3,10]. These phytochemicals possess notable antioxidant capabilities, which are essential for protecting against oxidative stress, a major contributor to cancer development [8,12]. The capacity of *A. racemosus* to augment antioxidant enzyme activity, such as superoxide dismutase (SOD) and catalase (CAT), further underscores its potential as a protective agent against cellular damage [11,13]. Moreover, the immunomodulatory effects of this herb may also contribute to its anticancer properties by enhancing the immune response of the body against tumor cells [14,15].

The specific mechanisms by which *A. racemosus* exerts its anticancer effects are under investigation. One example is the observed ability of plant extracts to induce apoptosis and inhibit cell proliferation in cancer cells [2,16]. Additionally, the modulation of various signaling pathways involved in cell survival and apoptosis presents a potential mechanism for its anticancer activity [3,17]. Given the multifaceted nature of TNBC and its resistance to conventional therapies, the exploration of *A. racemosus* as a complementary treatment option warrants further investigation.

Building on the promising pharmacological profile of *A. racemosus*, this study aims to investigate its anticancer potential specifically against human TNBC cells (MDA-MB-231). To identify key molecular targets, predict and validate bioactive compound binding, and evaluate anticancer mechanisms including cytotoxicity and apoptosis, we employ an integrated approach encompassing network pharmacology, molecular docking and dynamics, and in vitro assays. By combining computational and experimental methods, this study provides valuable insights into the therapeutic potential of *A. racemosus*, which might be helpful for its development as a novel, plant-based intervention for TNBC management.

## 2. Results

### 2.1. Screening of Phytochemical Compounds of A. racemosus

From the literature, a total of 47 different phytochemical constituents of *A. racemosus* were identified, which are used in this study. The drug-likeness characteristics of screened phytoconstituents were estimated via swissADME and the Molsoft web server. The results are presented in Supplementary Table S1. The initial screening revealed that six (stigmasterol, quercetin, kaempferol, beta-sitosterol-beta-D-glucoside, beta-sitosterol, and racemosol) out of the 47 compounds exhibited favorable drug-likeness properties (Table 1). Further analysis focused on these six compounds, as the remaining 41 compounds were excluded due to suboptimal drug-likeness (DL < 0.18) and bioavailability (BA < 0.30) scores.

### 2.2. Prediction of Targets and Screening for Potential Targets

**Figure 1:**
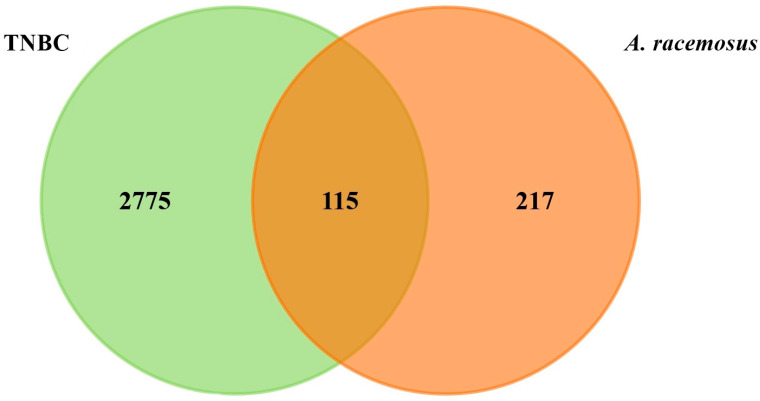
Venn diagram representing common targets between TNBC and *A. racemosus*.

The target classes of these compounds were analyzed using the SwissTargetPrediction database, while the PubChem database provided comprehensive information about them. For the phytochemical components of *A. racemosus*, a total of 332 projected targets were identified from the SwissTargetPrediction database, whereas 2890 predicted targets for TNBC were gathered from GeneCards (GDA cutoff > 30), DisGeNET (score cutoff > 0.1), and OMIM databases, with duplicates removed. FunRich was then used to identify common targets between the compounds and the disease, resulting in a total of 115 potential targets (Figure 1).

### 2.3. Identifying Hub Genes and Construction of Compounds-Disease Common Target Network

**Figure 2:**
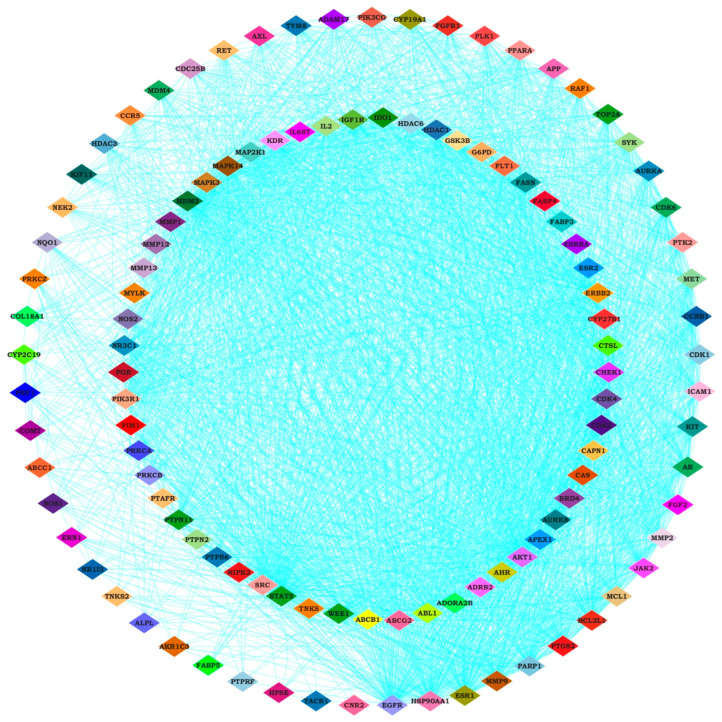
The network of common-gene targets of TNBC and phytochemical constituents of *A. racemosus* using Cytoscape software.

**Figure 3:**
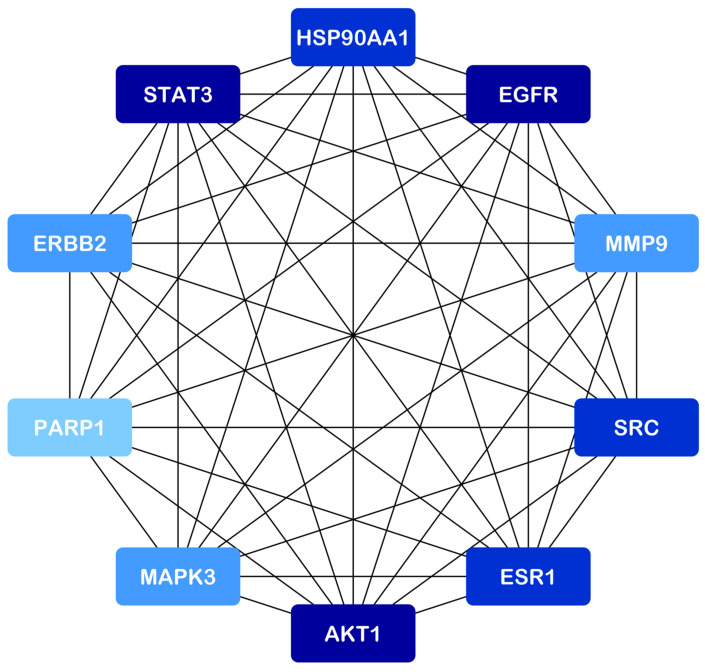
Identified hub-gene in a PPI network obtained from a common target gene of phytochemical constituents of *A. racemosus* and TNBC.

Protein–protein interaction (PPI) data for potential target genes were retrieved from the STRING database. PPI network visualization and analysis of this data were performed using Cytoscape (v3.10.1). Figure 2 represents the resulting common gene network. Degree, closeness, and betweenness centrality (calculated by cytoNCA) were used to assess node significance. These parameters reflect the importance of each node within the network. The identified target genes are known to be implicated in TNBC progression, suggesting that the anticancer activity of *A. racemosus* phytochemicals may involve these key targets. Then, we utilized the cytoHubba plugin in Cytoscape, a widely used tool for network analysis. Among the various available algorithms, we applied the degree centrality method, which ranks nodes based on the number of direct connections they have within the constructed compound–disease common target network. The top 10 genes with the highest degree values were identified as STAT3, EGFR, AKT1, ESR1, SRC, HSP90AA1, MAPK3, ERBB2, MMP9, and PARP1 (Figure 3). These top ten targets may represent the key molecular targets modulated by *A. racemosus* phytochemicals in the potential prevention and treatment of TNBC. The findings suggest that these hub genes could serve as promising therapeutic targets for TNBC, some of which are already being explored in existing or emerging drug therapies.

### 2.4. Analysis of Pathways and Functional Enrichment

**Figure 4:**
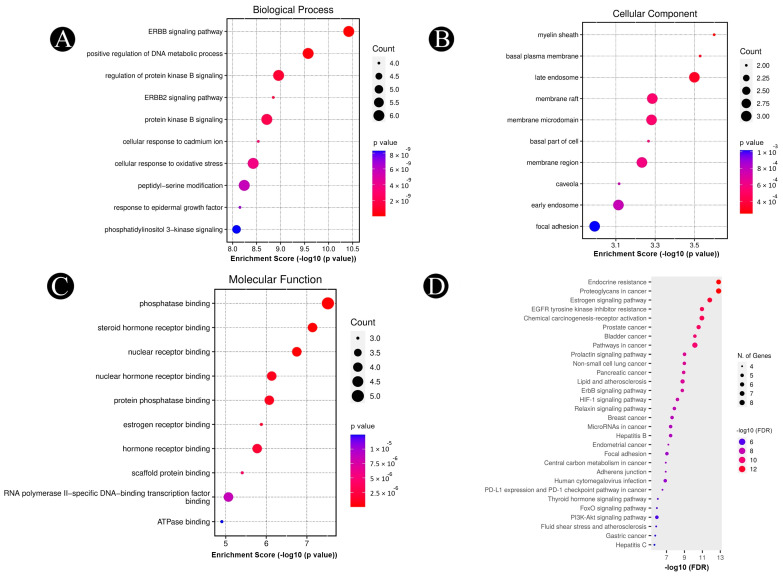
GO enrichment and KEGG pathway analyses of identified target proteins (*p*-value < 0.05). (**A**) The top 10 biological processes, (**B**) the top 10 cellular components, (**C**) the top 10 molecular functions, and (**D**) the top 10 KEGG pathways. The color scales indicate the different thresholds for the *p*-value, and the sizes of the dots represent the number of genes corresponding to each term.

The DAVID database was used to identify the target genes for triple-negative breast cancer. These genes were then further investigated to learn more about their roles, pathways, and involvement in the disease process. This would contribute to gaining a further understanding of the disease’s general process. Numerous GO enrichment items that were connected to the target genes were shown by the GO annotations. A total of 1510 BPs, 76 CCs, and 102 MFs were found. The top twenty enriched phrases for each KEGG pathway and the top ten in each GO category were displayed using a bubble chart (Figure 4A–D). These target genes are involved in biological processes, such as ERBB and ERBB2 signaling, positive regulation of DNA metabolic process, regulation of protein kinase B signaling, cellular response to oxidative stress, peptidyl-serine modification, response to epidermal growth factor and phosphatidylinositol 3-kinase signaling via different cellular compartments such as myelin sheath, basal plasma membrane, late endosome, membrane raft, membrane microdomain, basal part of the cell, caveola, early endosome, and focal adhesion via molecular functions, including phosphate binding, steroid hormone receptor binding, nuclear receptor binding, nuclear hormone receptor binding, protein phosphatase binding, estrogen receptor binding, scaffold protein binding, RNA polymerase II-specific DNA-binding transcription factor binding, and ATPase binding. There were a total of 151 KEGG pathways related to the identified hub genes. Key enriched pathways included proteoglycans in cancer, estrogen signaling, EGFR tyrosine kinase inhibitor resistance, prolactin signaling, ErbB signaling, HIF-1 signaling, PD-L1 expression and PD-1 checkpoint, FoxO signaling, PI3K-Akt signaling, pathways in cancer, prostate cancer, bladder cancer, pancreatic cancer, gastric cancer, and breast cancer. These findings suggest that the phytochemicals present in *A. racemosus* may have the ability to modulate the signaling pathways involved in TNBC.

### 2.5. Binding Affinity and Molecular Interaction Analysis

**Figure 5:**
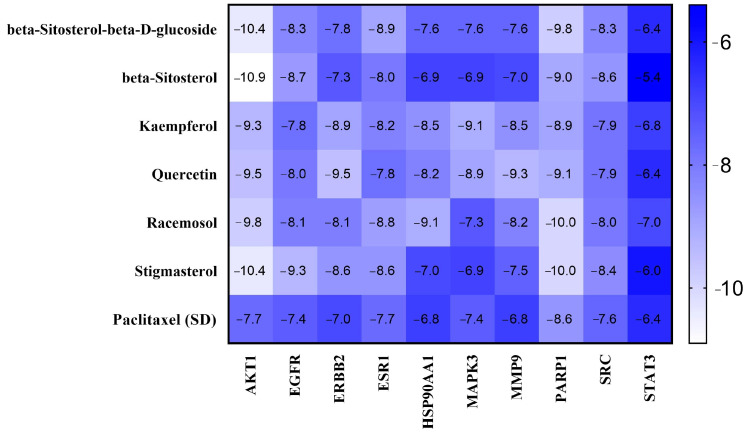
Binding affinities of top-rated pose of ligand–receptor complex.

Molecular docking analysis of *A. racemosus* phytochemicals against TNBC targets is presented in Figure 5. These binding energies reflect the stability of the interactions between the phytochemicals and target proteins, with lower binding energies indicating stronger, more stable binding. Several phytochemicals displayed significant binding affinity to specific protein targets.

**Figure 6:**
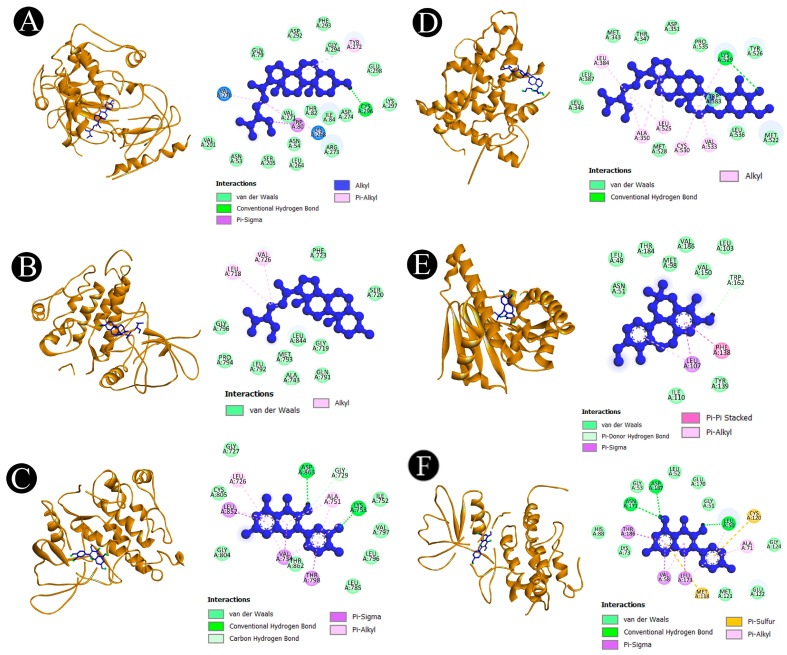
Visualization of the docking analysis. (**A**) Interaction of beta-sitosterol with AKT, (**B**) interaction of stigmasterol with EGFR, (**C**) interaction of quercetin with ERBB2, (**D**) interaction of beta-sitosterol-beta-D-glucoside with ESR1, (**E**) interaction of racemosol with HSP90, and (**F**) interaction of kaempferol with MAPK3. The light blue circular regions over amino acid residues represent solvent-accessible surfaces.

**Figure 7:**
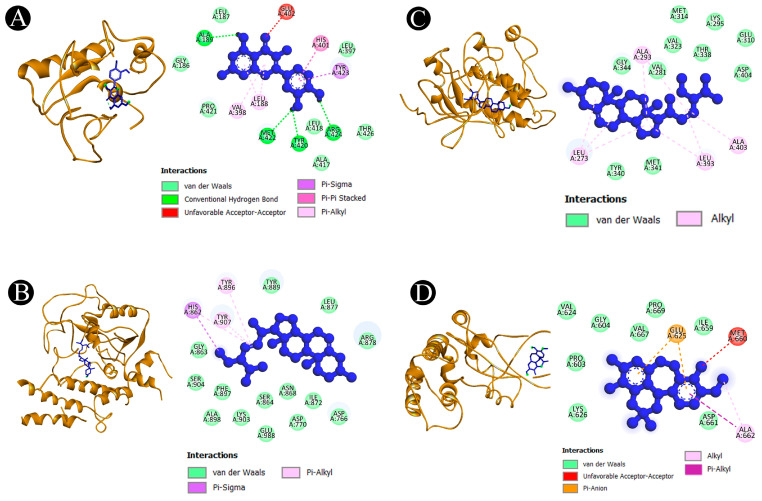
Visualization of the docking analysis. (**A**) Interaction of quercetin with MMP9, (**B**) interaction of stigmasterol with PARP1, (**C**) interaction of beta-sitosterol with SRC, and (**D**) interaction of racemosol with STAT3. The light blue circular regions over amino acid residues represent solvent-accessible surfaces.

The results of beta-sitosterol-beta-D-glucoside show highest binding energies towards AKT1 (−10.4 kcal/mol), beta-sitosterol shows the highest binding energies towards AKT1 (−10.9 kcal/mol), kaempferol shows the highest binding energies towards AKT1 (−10.4 kcal/mol), quercetin shows the highest binding energies towards AKT1 (−9.5 kcal/mol) and ERBB2 (−9.5 kcal/mol), racemosol shows the highest binding energies towards PARP1 (−10.0 kcal/mol), stigmasterol shows the highest binding energies towards AKT1 (−10.4 kcal/mol), and a standard drug paclitaxel shows the highest binding energies towards AKT1 (−7.7 kcal/mol) and ESR1 (−7.7 kcal/mol). Figure 6A–F and Figure 7A–D along with Supplementary Table S2 provide a thorough analysis of associations between *A. racemosus* phytochemical constituents and their respective target proteins.

### 2.6. MD Simulation Analysis

**Figure 8:**
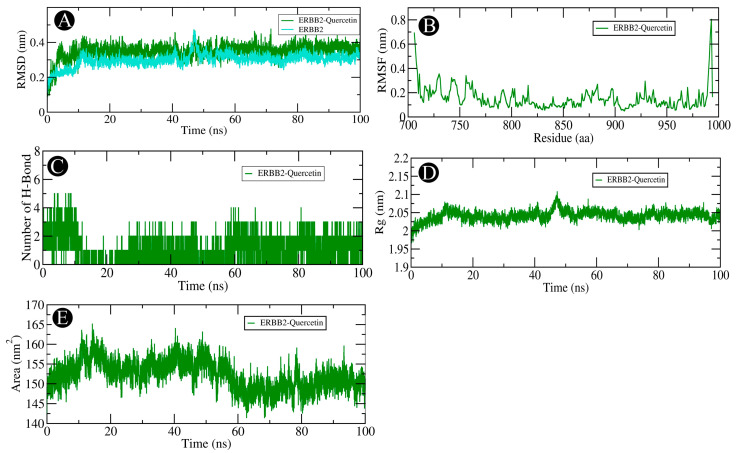
Analysis of the ERBB2-quercetin complex using molecular dynamics (MD) simulations. (**A**) RMSD over the simulation timescale. (**B**) RMSF of protein residues. (**C**) assessment of intermolecular hydrogen bond formation. (**D**) Rg distribution, reflecting protein compactness. (**E**) SASA plot, illustrating changes in protein folding.

**Figure 9:**
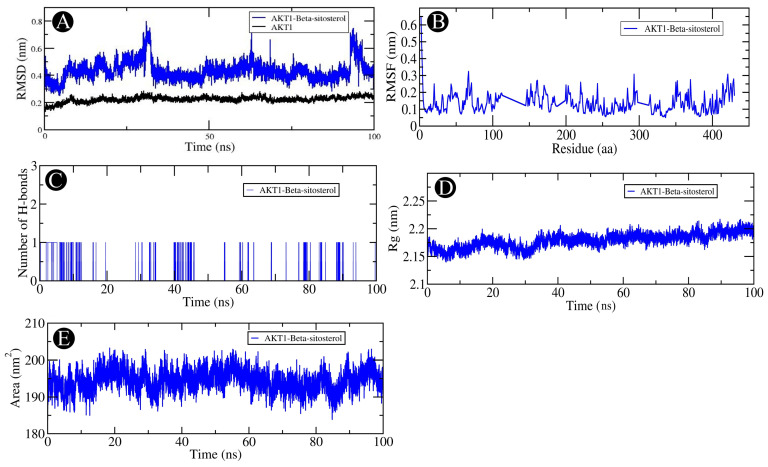
Analysis of the AKT1-beta-sitosterol complex using molecular dynamics (MD) simulations. (**A**) RMSD over the simulation timescale. (**B**) RMSF of protein residues. (**C**) Assessment of intermolecular hydrogen bond formation. (**D**) Rg distribution, reflecting protein compactness. (**E**) SASA plot, illustrating changes in protein folding.

Molecular dynamics (MD) simulations of 100 ns duration were performed to evaluate the stability of ERBB2-quercetin and AKT1-beta-sitosterol complexes. RMSD analysis showed stable conformations for both complexes, with ERBB2-quercetin exhibiting slightly higher fluctuations (0.3–0.4 nm) compared to unbound ERBB2 (0.2–0.3 nm) (Figure 8A) and AKT1-beta-sitosterol fluctuating between 0.3 and 0.8 nm compared to unbound AKT1 at 0.2–0.3 nm (Figure 9A). RMSF analysis revealed stable binding interactions for both complexes, with low fluctuations in the binding site regions, though some flexibility was observed in terminal and loop regions (Figure 8B and Figure 9B). Hydrogen bond analysis indicated dynamic hydrogen bond formation in ERBB2-quercetin, suggesting a recurring stabilizing interplay (Figure 8C), while AKT1-beta-sitosterol showed limited and transient hydrogen bond interactions (Figure 9C). The radius of gyration (Rg) remained stable for both complexes, with ERBB2-quercetin fluctuating between 2.0 and 2.1 nm (Figure 8D) and AKT1-beta-sitosterol between 2.15 and 2.25 nm (Figure 9D), indicating consistent, compact structures. Solvent accessible surface area (SASA) analysis showed fluctuations for both complexes (ERBB2-quercetin: 145–165 nm^2^; AKT1-beta-sitosterol: 190–205 nm^2^), suggesting dynamic solvent exposure due to side chain movements, but no major structural changes (Figure 8E and Figure 9E).

### 2.7. Anticancer Activity of A. racemosus Crude Extract

**Figure 10:**
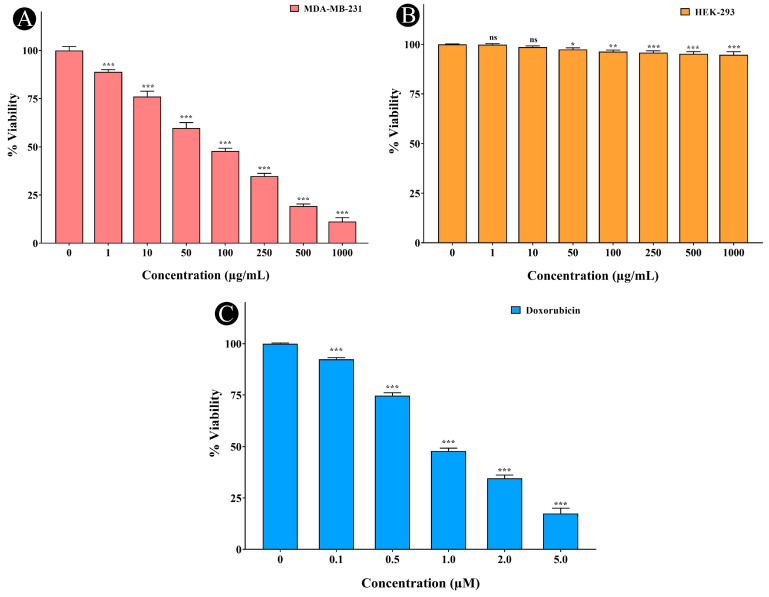
Determination of the cytotoxic effects of *A. racemosus* crude extract and the standard drug doxorubicin using the MTT assay. (**A**) Cell viability (%) of MDA-MB-231 cells after exposure to different concentrations of *A. racemosus* crude extract. (**B**) Cell viability (%) of HEK-293 cells after exposure to different concentrations of *A. racemosus* crude extract. (**C**) Cell viability (%) of MDA-MB-231 cells after exposure to different concentrations of doxorubicin. Data represent the mean ± SD of three independent experiments. Statistical significance: ns > 0.05, * *p* < 0.05, ** *p* < 0.005, *** *p* < 0.0005.

**Figure 11:**
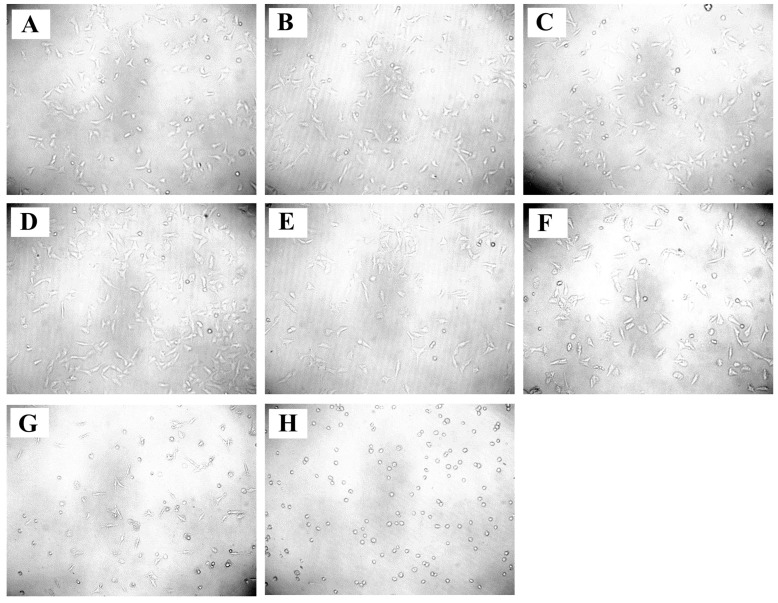
Morphological analysis of MDA-MB-231 cells under an inverted microscope after treatment with different concentrations of *A. racemosus* crude extract with morphological changes. (**A**) Untreated control, (**B**–**H**) representative images of cells treated with *A. racemosus* at (**B**) 1 μg/mL, (**C**) 10 μg/mL, (**D**) 50 μg/mL, (**E**) 100 μg/mL, (**F**) 250 μg/mL, (**G**) 500 μg/mL, and (**H**) 1000 μg/mL. Scale bar = 100 μM.

The MTT assay was employed to evaluate the anticancer activity of *A. racemosus* crude extract against MDA-MB-231 and HEK-293 cells. As shown in Figure 10A–C, a clear dose-dependent decrease in cell viability was observed against MDA-MB-231 cells. Higher concentrations of the extract corresponded to a greater reduction in the number of viable MDA-MB-231 cells. The IC_50_ value, determined to be 90.44 μg/mL against MDA-MB-231 (Figure 10A), indicates the concentration of the extract needed to reduce cell viability by 50%. A very low or negligible decrease in cell viability was observed when the plant extract was tested against HEK-293 cells, even at higher concentrations (Figure 10B). In contrast, the standard drug doxorubicin (0.1 to 5 µM) exhibited a significant reduction in cell viability even at low concentrations, with an IC₅₀ value of 1.0 µM (Figure 10C). Likewise, to further confirm the inhibitory effects of *A. racemosus* crude extract on cell growth, an assessment of its impact on cell confluency using an inverted microscope was carried out. Treatment with increasing extract concentrations gradually decreased the confluency of the MDA-MB-231 cell monolayer, as shown in Figure 11A–H. Additionally, treated cells exhibited morphological changes characterized by cell rounding, shrinkage, loss of alignment, and increased intercellular spaces, further supporting growth inhibitory properties of the extract.

### 2.8. Impact on Cell Cycle Regulation 

**Figure 12:**
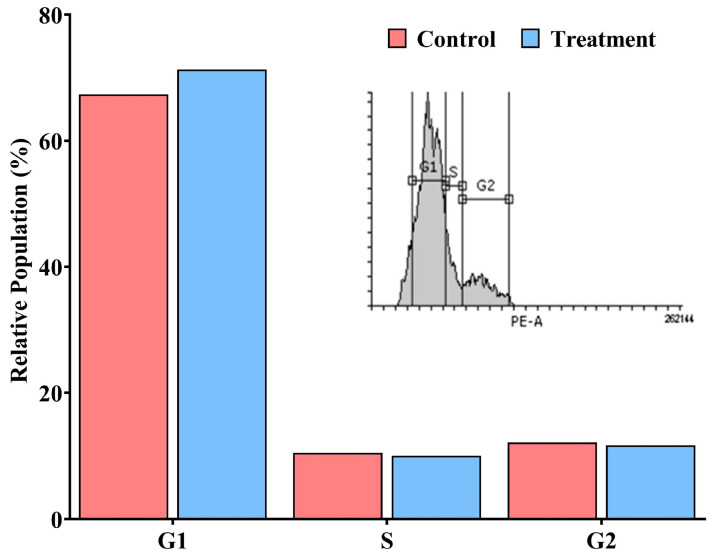
Cell cycle analysis of MDA-MB-231 cells. Comparison of untreated control and *A. racemosus* crude extract treatment at the IC_50_ concentration. The graph depicts the average percentage of cells in each cell cycle phase (G1, S, and G2/M) for both groups.

Flow cytometry was employed to analyze the effect of *A. racemosus* extract on the cell cycle distribution of MDA-MB-231 cells. Treatment with the extract at its IC_50_ concentration induced a prominent G1 phase arrest. Specifically, compared to the untreated control, the treated cells exhibited a significant increase in the percentage of cells in the G1 phase. Conversely, the proportion of cells in the S and G2 phases was reduced slightly following treatment. These results indicate that the *A. racemosus* extract exerts its effects on MDA-MB-231 cells by primarily disrupting progression through the cell cycle at the G1 checkpoint (Figure 12).

### 2.9. Detection of Apoptosis Using Propidium Iodide (PI) Staining with Annexin V

**Figure 13:**
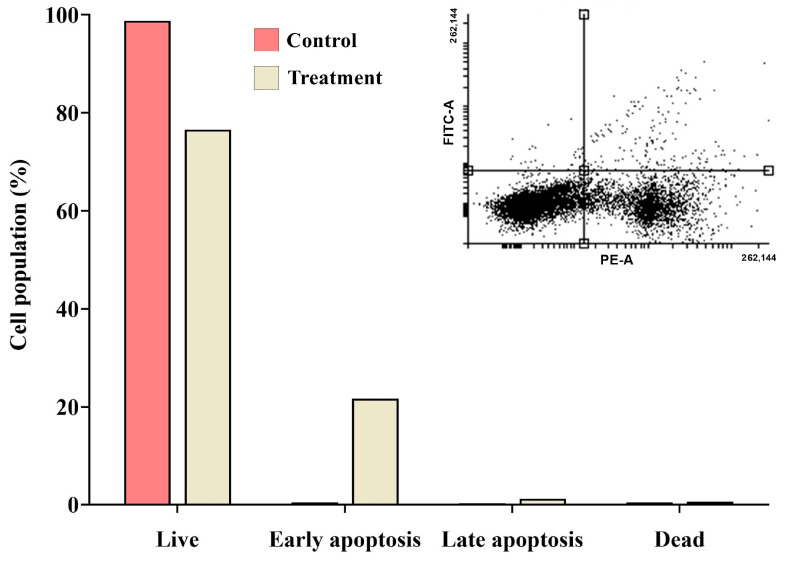
Assessment of apoptosis induction in MDA-MB-231 cells treated with *A. racemosus* crude extract (IC_50_) for 24 h. The Annexin V-PI staining assay was employed to quantify live, early, and late apoptotic and necrotic cell populations.

Apoptosis induction was assessed via Annexin V-FITC and propidium iodide (PI) staining, which revealed the distribution of MDA-MB-231 cells after treatment with the IC_50_ concentration of *A. racemosus* crude extract as a predominant population of live cells (76.60%), a substantial proportion of cells in early apoptosis (21.70%), a small percentage in late apoptosis (1.20%), and a very minor population of necrotic cells (0.50%). These results indicate that treatment with the *A. racemosus* extract primarily induces early apoptosis in MDA-MB-231 cells, with a smaller fraction progressing to late apoptosis, while necrosis appears to be a negligible factor at the tested concentration (Figure 13).

## 3. Discussion

This study highlights a comprehensive evaluation of the anticancer ability of *A. racemosus* against human TNBC, integrating in silico and in vitro approaches. Our network pharmacology analysis identified key pathways and potential targets modulated by *A. racemosus* phytochemicals, offering a valuable overview of the complex mechanism of action of plants. Network pharmacology analysis identified 115 potential targets shared between *A. racemosus* phytochemicals and TNBC, revealing an involvement in key cancer-related pathways, including ERBB/ERBB2 signaling, PI3K-Akt signaling, and pathways in cancer. Ten hub genes central to TNBC progression (STAT3, EGFR, AKT1, ESR1, SRC, HSP90AA1, MAPK3, ERBB2, MMP9, and PARP1) were identified, suggesting that *A. racemosus* may exert its effects by modulating these key regulators. Subsequent GO and KEGG pathway enrichment analysis further supported the involvement of these targets in crucial biological processes and signaling pathways relevant to TNBC development and progression.

The ERBB/ERBB2 signaling pathway and the PI3K-Akt signaling pathway are critical components in cancer biology, particularly in targeted therapies. They are important for cell proliferation, survival, and tumorigenesis, making them pivotal targets for cancer treatment. The ERBB family of receptors, particularly ERBB2 (also known as HER2), has been extensively studied due to its association with aggressive forms of breast cancer and other malignancies. ERBB2 functions as a coreceptor in the ERBB signaling network, which includes ERBB1, ERBB3, and ERBB4. Activation of this receptor family, initiated by ligand binding and dimerization, triggers downstream signaling pathways, including PI3K-Akt and MAPK [18,19]. Overexpression of ERBB2, a key driver of cell proliferation and survival, is associated with poor breast cancer outcomes. Targeted therapies, such as trastuzumab which inhibits this pathway, have demonstrated significant clinical benefit in ERBB2-positive breast cancers [18,20].

The PI3K-Akt signaling pathway is another important player in cancer biology. It is well-established that this pathway is associated with various cellular processes, including growth, metabolism, and survival [21,22]. Mutations in the PIK3CA gene or loss of PTEN function are common causes of dysregulation in this pathway in cancer, leading to increased cell survival and proliferation, which contributes to tumor progression and resistance to treatment [23,24]. Resistance to ERBB2-targeted therapies, often mediated by the PI3K-Akt pathway, underscores the clinical significance of this pathway. Compensatory PI3K-Akt activation, which can occur after ERBB2 inhibition, is one mechanism contributing to treatment failure [24,25]. Moreover, the interplay between ERBB2 and the PI3K-Akt pathway is complex and multifaceted. The reported studies indicate that ERBB2 can activate the PI3K-Akt pathway through various mechanisms, including heterodimerization with other ERBB family members, which enhances the signaling cascade [19,26]. This crosstalk not only promotes tumor cell survival but also facilitates the development of metastatic potential in various cancers, including breast and prostate cancer [26,27].

Molecular docking studies predicted strong binding affinities between specific *A. racemosus* phytochemicals and these identified TNBC targets. Notably, beta-sitosterol and its glucoside derivative, along with kaempferol and stigmasterol, exhibited high binding energies towards AKT1, while quercetin demonstrated strong interactions with both AKT1 and ERBB2. Racemosol showed a high affinity for PARP1. These findings suggest a direct interaction between these phytochemicals and key TNBC-related proteins, potentially disrupting their normal function. Molecular dynamics simulations of the ERBB2-quercetin and AKT1-beta-sitosterol complexes further validated these interactions, demonstrating stable complex formation over 100 ns. RMSF analysis confirmed stable binding site interactions, while hydrogen bond analysis revealed dynamic but recurring hydrogen bond formation in ERBB2-quercetin and limited transient interactions in AKT1-beta-sitosterol. Rg and SASA analysis indicated consistent, compact structures and dynamic solvent exposure, respectively, for both complexes, further supporting stable interactions. Beta-sitosterol is a prominent phytosterol that has shown significant anticancer properties across multiple cancer types, including breast cancer. Reported studies indicate that β-sitosterol can induce apoptosis in breast cancer cells by activating caspase-3 and -9, leading to mitochondrial dysfunction and cell cycle arrest [28,29]. Beta-sitosterol treatment induces G1 phase cell cycle arrest in MDA-MB-231 breast cancer cells by downregulating cyclin D1 and upregulating pro-apoptotic proteins like BAX [29,30]. Beta-sitosterol also inhibits the PI3K/Akt pathway, often dysregulated in cancer, leading to increased apoptosis and reduced cell proliferation [31]. Kaempferol, a flavonoid found in various plants, has also been implicated in breast cancer treatment. It exhibits potent antioxidant properties and has been shown to inhibit the proliferation of breast cancer cells through multiple mechanisms, including the modulation of cell cycle regulators and induction of apoptosis [32]. Stigmasterol, another phytosterol, exhibits anticancer activity by inducing endoplasmic reticulum (ER) stress and mitochondrial dysfunction in cancer cells. Studies have shown that stigmasterol activates ER stress sensors, triggering apoptosis in breast cancer cells [33]. Quercetin, a widely studied flavonoid, exhibits anticancer properties, including inhibiting breast cancer cell proliferation by inducing apoptosis and downregulating anti-apoptotic proteins [32]. Racemosol, a lesser-known compound, has also been identified for its anticancer properties. Although specific studies on racemosol in breast cancer are limited, its general bioactivity suggests potential mechanisms similar to those of other phytochemicals, including the induction of apoptosis and modulation of cell signaling pathways [34].

The in vitro component of the present study provided crucial experimental evidence supporting the observed in silico predictions. The *A. racemosus* crude extract demonstrated a dose-dependent reduction in MDA-MB-231 cell viability, with an IC_50_ of 90.44 μg/mL, confirming its potent anticancer activity. Microscopic observations revealed decreased cell confluency and characteristic morphological changes indicative of growth inhibition, including cell rounding, shrinkage, and increased intercellular spaces. Flow cytometric cell cycle analysis demonstrated a significant G1 phase arrest upon treatment with the extract, suggesting a disruption in cell cycle progression at this critical checkpoint. Furthermore, Annexin V/PI staining revealed a substantial proportion of cells in early apoptosis (21.70%) following treatment, with smaller populations in late apoptosis (1.20%) and necrosis (0.50%), indicating that the extract induces programmed cell death, primarily via the early apoptotic pathway. One of the primary mechanisms by which *A. racemosus* exhibits its anticancer properties is via induction of the PI3K/Akt signaling pathway. This pathway is frequently dysregulated in several cancers, including breast cancer, leading to enhanced cell survival and proliferation [14].

The reported studies indicate that extracts from *A. racemosus* can inhibit the activation of this pathway, thereby promoting apoptosis in cancer cells. Furthermore, several compounds derived from *A. racemosus*, such as asparanin A, induce G0/G1 cell cycle arrest and apoptosis in cancer cell lines by targeting mitochondrial functions and the PI3K/Akt signaling cascade [35]. This suggests that *A. racemosus* may serve as a potential adjunct therapy in breast cancer treatment via modulating the efficacy of conventional therapies that target the PI3K/Akt pathway. Additionally, the presence of steroidal saponins in *A. racemosus* has been linked to its anticancer effects. These compounds exhibit cytotoxicity against various cancer cell lines, including breast cancer cells, through the induction of apoptosis and inhibition of cell proliferation [14]. The saponins may disrupt cellular membranes and interfere with intracellular signaling pathways, resulting in increased apoptosis and decreased tumor growth [14]. The elevated oxidative stress frequently observed in cancerous tissues can be mitigated by the antioxidant properties of these compounds, thus contributing to their overall anticancer effect [14]. Racemosol, another active constituent of *A. racemosus* has also been implicated in its anticancer activity via induction of apoptosis and modulation of cell signaling pathways [14]. The presence of isoflavones and other phytochemicals in *A. racemosus* further supports its role as a protective agent against cancer, as these compounds have been shown to exhibit estrogenic activity, which may be beneficial in hormone-sensitive breast cancers [14].

Overall, this study provides strong evidence for the anticancer potential of *A. racemosus* against TNBC. The combined in silico and in vitro data converge to support a mechanistic rationale for its activity, highlighting the modulation of key oncogenic pathways and targets, including AKT1 and ERBB2, as well as the induction of G1 arrest and apoptosis. These findings suggest that *A. racemosus* warrants additional research as a possible therapeutic agent for TNBC. Future studies could explore synergistic effects with existing chemotherapeutics, investigate specific mechanisms of action in greater detail, and evaluate efficacy in in vivo models.

## 4. Materials and Methods

### 4.1. In Silico Screening of Bioactive Molecules

Active constituents of *A. racemosus* were identified through a literature review supported by databases like IMPPAT (version 2.0) and KNApSAcK (version 4) [36]. Canonical SMILES and chemical structures were obtained for each identified compound. A key step in the process was the in silico evaluation of ADME-related properties, specifically the bioavailability (OB) and drug-likeness (DL). Only compounds exhibiting both a DL greater than 0.18 and an OB of 30% or higher were considered suitable for subsequent analysis. Compounds failing to meet these ADME criteria were not progressed further. Molsoft and SwissADME were the computational tools used for these assessments [37].

### 4.2. Exploring Targets in Compound-Disease Relationships

Phytochemicals documented in the literature for *A. racemosus* were analyzed by retrieving their SMILES codes from the PubChem database. These SMILES strings were then used as input for target prediction using the SwissTargetPrediction database (www.swisstargetprediction.ch, accessed on 15 January 2025). Genes associated with triple-negative breast cancer were identified through searches in GeneCards (https://www.genecards.org/, accessed on 15 January 2025) using the search term “triple negative breast cancer”. To further refine the target list, DisGeNET (http://www.disgenet.org/, accessed on 15 January 2025) and OMIM (https://www.omim.org/, accessed on 15 January 2025) databases were used. A DisGeNET score threshold of >0.1 and a GeneCards relevance score threshold of >30 [38] were applied for target selection.

### 4.3. Identifying and Obtaining Potential Targets

Shared targets between *A. racemosus* phytochemicals and TNBC were identified using the FunRich tool (version 3.1.3). SwissTargetPrediction was employed to further classify the potential protein targets. Venn diagrams visualizing these shared targets were generated using FunRich [39].

### 4.4. Hub Gene Identification and Protein Interaction Network Analysis

Hub gene identification was performed using the cytoHubba plugin within Cytoscape (version 3.10.1), selecting the top ten based on degree centrality. Protein–protein interaction (PPI) data for predicted target proteins were retrieved from the STRING database (accessed on 20 January 2025) [40]. Network data from STRING were imported into Cytoscape for PPI network construction and analysis. A false discovery rate (FDR) of 5% and a confidence score of 0.400 were used as data analysis parameters. Degree, betweenness centrality, and closeness centrality were calculated to characterize network nodes and guide target selection.

### 4.5. GO and KEGG Pathway Enrichment Analysis

The DAVID database (https://david.ncifcrf.gov/, accessed on 20 January 2025) was used for functional enrichment and pathway analysis of target proteins and the associated disease [41]. A false discovery rate (FDR) cutoff of 0.05 was used for enrichment analysis, with results presented as Gene Ontology (GO) terms and pathways. SRplot (https://www.bioinformatics.com.cn/, accessed on 20 January 2025) generated a bubble plot visualizing the top ten significant GO terms (biological process, cellular component, and molecular function). ShinyGo 0.77 (http://bioinformatics.sdstate.edu/go/, accessed on 20 January 2025) mapped the top twenty KEGG pathways.

### 4.6. Molecular Docking Studies

AutoDock v1.5.7 was used to assess the phytochemical contents of *A. racemosus* binding affinity to TNBC targets [42]. Ligand 3D structures were obtained from PubChem [43] and converted from .sdf to .pdb format using Open Babel v2.4.1. Energy minimization was performed using Avogadro (version 1.2.0) with the MMFF94 force field and 5000 steps of steepest descent, terminating when the energy change was below 0.1. The minimized structures were saved as .pdb files. From the RCSB Protein Data Bank database [44], the 3D crystal structures by X-Ray Diffraction technique were downloaded with the following PDB-ID, AKT1 (PDB ID:3O96), EGFR (PDB ID:4ZAU), ERBB2 (PDB ID:3PP0), ESR1 (PDB ID:3ERT), HSP90AA1 (PDB ID:5H22), MAPK3 (PDB ID:3FHR), MMP9 (PDB ID:1GKC), PARP1 (PDB ID:7KK3), SRC (PDB ID:4MX0) and STAT3 (PDB ID:6NJS). Polar hydrogens and Kollman charges were introduced to the receptors after the water molecules and heteroatoms were removed. Subsequently, the protein structures were saved as .pdbqt files. The protein–ligand complexes’ active sites were retrieved from the CASTp server and docked using AutoDock Vina (version 1.1.2) [45] with an exhaustiveness of 8, and the coordinates of every receptor. PyMoL (version 2.5.2) [46] and Biovia Discovery Studio Visualizer (version 21.1.0.20298) [47] were then used to view the results.

### 4.7. Molecular Dynamics (MD) Simulation

The stability of ligand conformation within the receptor binding pocket over time was investigated using MD simulations, a technique with demonstrated utility in identifying novel inhibitors [38,48]. GROMACS (version 2019.4) [49] was the chosen MD engine, employing the CHARMM36m force field. The process began with ligand topology generation using SwissParam. An initial energy minimization was performed in a vacuum using the steepest descent algorithm for 1500 steps. The resulting system was then solvated in a cubic box (0.5 nm) of TIP3P water, and Na+ and Cl- ions were added to neutralize the system and achieve a 0.15 M salt concentration. After 100 ps of NVT and NPT equilibration, a 100 ns production MD simulation was conducted. Periodic boundary conditions were removed from the trajectory file during post-processing. Trajectory analysis was performed using Chimera, and XMGRACE (https://plasma-gate.weizmann.ac.il/Grace/, accessed on 28 January 2025) was used to create graphical representations of the data.

### 4.8. Plant Material Collection and Preparation of Extract

The preparation of the *A. racemosus* extract involved several steps. Aerial parts of the plant were collected from the Bapalal Vaidya Botanical Research Centre (Surat, Gujarat, India) and subsequently dried and ground into a fine powder. The powdered material (10 g) was macerated in 200 mL of distilled water and allowed to stand overnight at room temperature. The resulting aqueous extract was then clarified by filtration through a Whatman No. 1 paper. Following filtration, the extract was concentrated using a rotary evaporator. The concentrated extract was subsequently dried in a 45 °C water bath until a stable weight was achieved [50].

### 4.9. Culturing of Cells

MDA-MB-231 cells, originating from a human triple-negative breast cancer patient, were derived from the National Centre for Cell Science (NCCS), Pune, India. Cells (MDA-MB-231) were grown in DMEM supplemented with 10% FBS, 10,000 units of penicillin, and 5 mg of streptomycin. The culture was maintained at 37 °C under a humidified atmosphere with 5% CO_2_ [51].

### 4.10. Cell Viability Study

Cell viability of TNBC cells treated with *A. racemosus* crude extract was evaluated using the MTT assay against MDA-MB-231 (triple-negative breast cancer) and HEK293 (human embryonic kidney) cells. Cells were trypsinized, aspirated, and centrifuged (3000 rpm, 5 min) from T-25 flasks. The resulting cell pellet was resuspended in growth media to a cell density of approximately 10,000 cells per 200 μL. Then, 200 μL of this suspension was seeded into each well of a 96-well plate and incubated for 24 h at 37 °C and 5% CO_2_ for cell attachment. Following the removal of the spent medium, MDA-MB-231 cells were treated with 200 μL of varying concentrations (1, 10, 100, 250, 500, and 1000 μg/mL) of *A. racemosus* crude extract for 24 h under standard culture conditions. After this incubation period, 200 μL of fresh medium containing 10% MTT reagent was added to each well, and the cells were incubated for an additional 3 h. Subsequently, 100 μL of DMSO was added to each well, and the plates were gently agitated to dissolve any formed formazan crystals. Absorbance was then measured at 570 nm and 630 nm using a microplate reader (BioTek Instruments, Agilent Technologies, Santa Clara, CA, USA). Doxorubicin (0.5 to 2 µM) was used as a positive control in this assay. The IC_50_ value was calculated after subtracting background and blank readings from the experimental readings [52].

### 4.11. Cell Cycle Analysis

MDA-MB-231 cells, seeded as previously described, were treated with *A. racemosus* extract at its IC_50_ concentration for 24 h. After the incubation period, the cells were detached from the culture vessel using trypsin and collected in 1.5 mL microcentrifuge tubes. The cells were washed once with 500 µL of ice-cold PBS. To create a uniform single-cell suspension and minimize cell clumping, approximately 1 × 10⁶ cells were resuspended in 100 µL of PBS and gently vortexed. This resulting suspension was then added to tubes containing ice-cold 70% ethanol and incubated for 2 h to fix the cells. Following fixation, the cells were centrifuged, and the resulting cell pellet was resuspended in a staining solution (prepared in PBS) containing 10 µg/mL propidium iodide (PI), 0.1% *v*/*v* Triton X-100, and 100 µg/mL RNase A. The cells were then incubated in the dark at room temperature for 30 min before undergoing analysis by flow cytometry [53].

### 4.12. Annexin-V Apoptosis Assay

The effect of *A. racemosus* extract on apoptosis in MDA-MB-231 cells was examined. MDA-MB-231 cells (5 × 10^4^ cells per 2 mL) were seeded in 6-well plates and incubated for 24 h at 37 °C in a CO₂ incubator. Following incubation, the spent medium was removed and the cells were washed with 1 mL of ice-cold phosphate-buffered saline (PBS). The cells were then treated with *A. racemosus* extract at its IC_50_ concentration for 24 h in 2 mL of growth media. One well, left untreated, served as the negative control. After two washes with cold PBS, the cells were resuspended in 1X binding buffer at a concentration of 1 × 10⁶ cells/mL. The cells were then divided into five groups: a treatment group, a propidium iodide (PI)-only group, an Annexin V-FITC-only group, a control group (untreated), and an unstained group. Annexin V-FITC and PI were added to the appropriately labeled tubes. After vortexing, 1 mL of 1X binding buffer was added to each tube, and the samples were incubated for 15 min at room temperature. The samples were subsequently analyzed using flow cytometry [54].

### 4.13. Statistical Analysis

The results of the MTT assay are presented as the mean ± SD of the number of experiments performed. The significance of the results was determined for the treatments using an ordinary one-way ANOVA followed by Dunnett’s multiple comparisons test at *p* < 0.05. The analyses were carried out using the Graph Pad Prism software 8.0.

## 5. Conclusions

This study provides compelling evidence for the anticancer potential of *A. racemosus* against TNBC through a combined in silico and in vitro approach. Our integrated methodology, including network pharmacology, molecular docking, molecular dynamics simulations, and a set of in vitro assays, has elucidated potential mechanisms underlying the observed anticancer effects. The in silico analyses identified key TNBC-related pathways and targets, including AKT1 and ERBB2, and predicted strong interactions between specific *A. racemosus* phytochemicals, notably beta-sitosterol and quercetin, with these crucial proteins. These computational findings were strongly supported by in vitro evidence demonstrating a dose-dependent reduction in TNBC cell viability, G1 phase cell cycle arrest, and the induction of early apoptosis upon treatment with *A. racemosus* extract. The observed correlation between in silico target prediction and in vitro functional assays strengthens the rationale for *A. racemosus* as a promising candidate for further development as a therapeutic strategy against TNBC. While further research, including in vivo studies and mechanistic investigations, is required, this study provides a strong foundation for exploring the clinical potential of A. racemosus in the fight against this aggressive form of breast cancer.

## Figures and Tables

**Table 1:** List of *A. racemosus* compounds exhibited favorable drug-likeness properties.

Compound Name	Pubchem ID	Molecular Formula	Molecular Weight (g/mol)	Drug Likeliness	Bioavailability Score	BBB Permeant	Structure
Stigmasterol	5280794	C_29_H_48_O	412.7	0.62	0.55	No	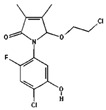
Quercetin	5280343	C_15_H_10_O_7_	302.23	0.52	0.55	No	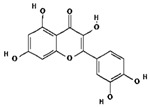
Kaempferol	5280863	C_15_H_10_O_6_	286.24	0.50	0.55	No	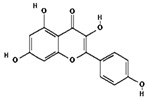
beta-Sitosterol- beta-D-glucoside	12309055	C_35_H_60_O_6_	576.8	0.5	0.55	No	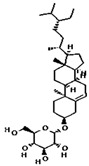
beta-Sitosterol	222284	C_29_H_50_O	414.7	0.78	0.55	No	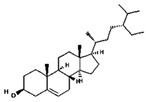
Racemosol	624971	C_21_H_24_O_4_	340.4	0.36	0.55	Yes	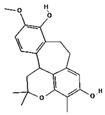

## Data Availability

The datasets generated during and/or analyzed during the current study are available from the corresponding author upon reasonable request due to privacy.

## Supplementary Materials

The following supporting information can be downloaded at: https://www.mdpi.com/article/10.3390/ph18030433/s1, Table S1: List of phytochemical constituents of *A. racemosus* passing bioavailability and drug-likeness properties; Table S2: Interacting active site residues of receptors and ligands.
