# Supplementary material for: Mechanistic Insights into the Anticancer Potential of Asparagus racemosus Willd. Against Triple-Negative Breast Cancer: A Network Pharmacology and Experimental Validation Study
Source: Pharmaceuticals (Basel). 2025 Mar 19;18(3):433. doi: 10.3390/ph18030433 (PMC11944961; doi:10.3390/ph18030433)
Supplement: Supplementary file 1 [file pharmaceuticals-18-00433-s001.zip › Table S1.pdf]

Supplementary Table S1. List of phytochemical constituents of *A. racemosus* passing bioavailability and drug-likeness properties.

Compound Name	Pubchem ID	Molecular Formula	Molecular Weight (g/mol)	Drug likeliness	Bioavailability Score	BBB Permeant
Stigmasterol	5280794	$C_{29}H_{48}O$	412.7	0.62	0.55	No
Shatavarin I	101406647	$C_{51}H_{86}O_{23}$	1067.2	0.33	0.17	No
Sarsasapogenin	92095	$C_{27}H_{44}O_3$	416.6	-0.74	0.55	Yes
Saccharin	5143	$C_7H_5NO_3S$	183.19	-0.86	0.55	No
Rutin	5280805	$C_{27}H_{30}O_{16}$	610.5	0.91	0.17	No
quercetin 3-O-glucuronide	5274585	$C_{21}H_{18}O_{13}$	478.4	0.81	0.11	No
Quercetin	5280343	$C_{15}H_{10}O_7$	302.23	0.52	0.55	No
L-Rhamnose	25310	$C_6H_{12}O_5$	164.16	-1.05	0.55	No
Kaempferol	5280863	$C_{15}H_{10}O_6$	286.24	0.50	0.55	No
Hyperoside	5281643	$C_{21}H_{20}O_{12}$	464.4	0.68	0.17	No
Diosgenin	99474	$C_{27}H_{42}O_3$	414.6	-0.09	0.55	Yes
D-Glucose	5793	$C_6H_{12}O_6$	180.16	-0.12	0.55	No
D-Galacturonic Acid	439215	$C_6H_{10}O_7$	194.14	-0.3	0.56	No
Cyanin	441688	$C_{27}H_{31}O_{16}$	611.5	0.35	0.17	No
β-Sitosterol-β-D-glucoside	12309055	$C_{35}H_{60}O_6$	576.8	0.5	0.55	No
β-Sitosterol	222284	$C_{29}H_{50}O$	414.7	0.78	0.55	No
Asparanin B	441896	$C_{45}H_{74}O_{17}$	887.1	-0.24	0.17	No
3-(Galactosyloxy)-3',4',5,7-tetrahydroxyflavylium chloride	176457	$C_{21}H_{21}ClO_{11}$	484.8	0.06	0.17	No
camphor	2537	$C_{10}H_{16}O$	152.23	0.11	0.55	Yes
limonene	22311	$C_{10}H_{16}$	136.23	-1.54	0.55	Yes
Isopentenyl diphosphate	1195	$C_5H_{12}O_7P_2$	246.09	-0.97	0.56	No
Chelidonic acid	7431	$C_7H_4O_6$	184.10	-0.97	0.56	No
vanillin	1183	$C_8H_8O_3$	152.15	-1.24	0.55	Yes

Compound Name	Pubchem ID	Molecular Formula	Molecular Weight (g/mol)	Drug likeliness	Bioavailability Score	BBB Permeant
borneol	64685	$C_{10}H_{18}O$	154.25	-0.51	0.55	Yes
Camphene	6616	$C_{10}H_{16}$	136.23	-1.32	0.55	Yes
Protodioscin	441891	$C_{51}H_{84}O_{22}$	1049.2	0.77	0.17	No
Cycloartenol	92110	$C_{30}H_{50}O$	426.7	-0.27	0.55	No
Hyperin	5281643	$C_{21}H_{20}O_{12}$	464.4	0.68	0.17	No
Cyanidin 3-O-β-D-galactopyranoside	441699	$C_{21}H_{21}O_{11}^{+}$	449.4	0.07	0.17	No
(2E,6E)-Farnesyl diphosphate	445713	$C_{15}H_{28}O_7P_2$	382.33	-0.97	0.56	No
(S)-2,3-Epoxysqualene	5459811	$C_{30}H_{50}O$	426.7	-0.92	0.55	No
Dimethylallyl diphosphate	647	$C_5H_{12}O_7P_2$	246.09	-0.97	0.56	No
trans-(-)-Pinocarveol	1201530	$C_{10}H_{16}O$	152.23	-1.51	0.55	Yes
cis-Verbenol	164888	$C_{10}H_{16}O$	152.23	-1.36	0.55	Yes
Pseudoprotodioscin	21637110	$C_{51}H_{82}O_{21}$	1031.2	0.89	0.17	No
(-)-Asparanin A	21575007	$C_{39}H_{64}O_{13}$	740.9	-0.25	0.17	No
5-Caranol	86056	$C_{10}H_{18}O$	154.25	-1.38	0.55	Yes
Aspafilioside C	148363	$C_{49}H_{82}O_{22}$	1023.2	0.16	0.17	No
Asparanin B5	163183853	$C_{50}H_{82}O_{21}$	1019.2	-0.32	0.17	No
Asparanin B9	163183852	$C_{50}H_{84}O_{23}$	1053.2	0.18	0.17	No
Asparaside A	163183846	$C_{51}H_{86}O_{23}$	1067.2	0.18	0.17	No
Carene	26049	$C_{10}H_{16}$	136.23	-1.47	0.55	Yes
Racemosol	624971	$C_{21}H_{24}O_4$	340.4	0.36	0.55	Yes
Shatavaroside A	44203608	$C_{44}H_{72}O_{16}$	857.0	-0.2	0.17	No
Shatavaroside B	44203607	$C_{50}H_{82}O_{21}$	1019.2	-0.3	0.17	No
Asparanin C	158604	$C_{44}H_{72}O_{16}$	857.0	-0.20	0.17	No
Filiasparoside C	44445742	$C_{44}H_{72}O_{15}$	841.0	-0.23	0.17	No
