# Supplementary material for: Mechanistic Insights into the Anticancer Potential of Asparagus racemosus Willd. Against Triple-Negative Breast Cancer: A Network Pharmacology and Experimental Validation Study
Source: Pharmaceuticals (Basel). 2025 Mar 19;18(3):433. doi: 10.3390/ph18030433 (PMC11944961; doi:10.3390/ph18030433)
Supplement: Supplementary file 1 [file pharmaceuticals-18-00433-s001.zip › Table S2.pdf]

Table S2. Interacting active site residues of receptors and ligands.

Protein-Ligand	Receptor-Ligand	Interaction Type	Distance
AKT-β-sitosterol	UNL1:H - A:CYS296:O	Conventional Hydrogen Bond	1.84533
	UNL1:C - A:TRP80	Pi-Sigma	3.5479
	A:LYS268 - :UNL1	Alkyl	5.46447
	A:VAL270 - :UNL1	Alkyl	4.13303
	A:TRP80 - :UNL1	Pi-Alkyl	4.33605
	A:TYR272 - :UNL1	Pi-Alkyl	5.37633
EGFR-stigmasterol	A:LEU718 - :UNL1	Alkyl	4.91726
	A:VAL726 - :UNL1	Alkyl	4.87084
ERBB2-qurcetine	A:LYS753:HN - :UNL1:O	Conventional Hydrogen Bond	2.68114
	:UNL1:H - A:ASP863:OD2	Conventional Hydrogen Bond	2.3666
	A:GLY729:CA - UNL1:O	Carbon Hydrogen Bond	3.75534
	A:VAL734:CG1 - :UNL1	Pi-Sigma	3.72842
	A:VAL734:CG2 - :UNL1	Pi-Sigma	3.8446
	A:THR798:CG2 - :UNL1	Pi-Sigma	3.83365
	A:LEU852:CD2 - :UNL1	Pi-Sigma	3.42461
	UNL1 - A:ALA751	Pi-Alkyl	5.35415
	UNL1 - A:LEU852	Pi-Alkyl	4.90812
	UNL1 - A:LEU726	Pi-Alkyl	4.91492
	UNL1 - A:VAL734	Pi-Alkyl	4.82933
	UNL1 - A:VAL734	Pi-Alkyl	5.00887
	UNL1 - A:ALA751	Pi-Alkyl	5.0504
	UNL1 - A:LYS753	Pi-Alkyl	4.21907
ESR1-β-sitosterol-β-D-glucoside	A:LYS529:HZ3 - UNL1:O	Conventional Hydrogen Bond	2.58669
	A:ALA350 - :UNL1	Alkyl	5.23864
	A:ALA350 - :UNL1	Alkyl	4.38629
	A:LEU384 - :UNL1	Alkyl	5.16023
	A:LEU525 - :UNL1	Alkyl	5.27375
	A:LEU525 - :UNL1	Alkyl	4.5747
	A:LYS529 - :UNL1	Alkyl	5.18672
	A:CYS530 - :UNL1	Alkyl	5.38511
	A:VAL533 - :UNL1	Alkyl	4.56124

Protein-Ligand	Receptor-Ligand	Interaction Type	Distance
HSP90-racemosol	UNL1:H - A:TRP162	Pi-Donor Hydrogen Bond	3.18229
	A:LEU107:CD1 - :UNL1	Pi-Sigma	3.81589
	A:LEU107:CD2 - :UNL1	Pi-Sigma	3.76088
	A:PHE138 - :UNL1	Pi-Pi Stacked	3.76315
	UNL1 - A:LEU107	Pi-Alkyl	5.37619
MAPK3-kaempferol	A:ASN171:ND2 - UNL1:O	Conventional Hydrogen Bond	3.27898
	UNL1:H - A:ASP187:OD1	Conventional Hydrogen Bond	2.58161
	UNL1:H - A:LEU50:O	Conventional Hydrogen Bond	2.24664
	A:VAL58:CG2 - :UNL1	Pi-Sigma	3.76238
	A:LEU173:CD2 - :UNL1	Pi-Sigma	3.74061
	A:THR186:CG2 - :UNL1	Pi-Sigma	3.57608
	A:MET118:SD - :UNL1	Pi-Sulfur	5.17213
	A:CYS120:SG - :UNL1	Pi-Sulfur	5.70095
	UNL1 - A:VAL58	Pi-Alkyl	4.13939
	UNL1 - A:LEU50	Pi-Alkyl	4.10488
	UNL1 - A:ALA71	Pi-Alkyl	4.9968
	UNL1 - A:LEU173	Pi-Alkyl	5.09326
MMP9-qurcetine	UNL1:H - A:TYR420:O	Conventional Hydrogen Bond	2.37635
	UNL1:H - A:MET422:O	Conventional Hydrogen Bond	2.644
	UNL1:H - A:ARG424:O	Conventional Hydrogen Bond	2.75755
	UNL1:H - A:ALA189:O	Conventional Hydrogen Bond	2.31508
	UNL1:H - :UNL1:O	Conventional Hydrogen Bond	1.91243
	A:TYR423:CA - :UNL1	Pi-Sigma	3.92404
	A:HIS401 - :UNL1	Pi-Pi Stacked	4.68657
	UNL1 - A:LEU188	Pi-Alkyl	4.64603
	UNL1 - A:VAL398	Pi-Alkyl	5.06729
	UNL1 - A:LEU188	Pi-Alkyl	4.43451
PARP1-stigmasterol	UNL1:C - A:HIS862	Pi-Sigma	3.89442
	A:HIS862 - :UNL1	Pi-Alkyl	5.06159
	A:TYR896 - :UNL1	Pi-Alkyl	5.15888
	A:TYR907 - :UNL1	Pi-Alkyl	4.34716

Protein-Ligand	Receptor-Ligand	Interaction Type	Distance
SRC-β-sitosterol	A:LEU273 - :UNL1	Alkyl	4.48897
	A:LEU273 - :UNL1	Alkyl	4.05868
	A:LEU273 - :UNL1	Alkyl	5.30418
	A:ALA293 - :UNL1	Alkyl	4.85015
	A:ALA293 - :UNL1	Alkyl	5.31628
	A:LEU393 - :UNL1	Alkyl	5.00374
	A:LEU393 - :UNL1	Alkyl	4.95499
	A:ALA403 - :UNL1	Alkyl	5.01108
STAT3-racemosol	A:GLU625:OE1 - :UNL1	Pi-Anion	4.02664
	A:GLU625:OE2 - :UNL1	Pi-Anion	3.58926
	A:ALA662 - :UNL1:C	Alkyl	4.05652
	UNL1 - A:ALA662	Pi-Alkyl	5.23757
